# Development and validation of a pragmatic measure of context at the organizational level: The Inventory of Factors Affecting Successful Implementation and Sustainment (IFASIS)

**DOI:** 10.1186/s43058-025-00726-9

**Published:** 2025-04-25

**Authors:** Hélène Chokron Garneau, Hannah Cheng, Jane Kim, Maryam Abdel Magid, Lia Chin-Purcell, Mark McGovern

**Affiliations:** Stanford Center for Dissemination and Implementation, Department of Psychiatry & Behavioral Sciences, Stanford University School of Medicine,, Palo Alto, CA USA

**Keywords:** Context, Instrument, Implementation Science, Generalizability

## Abstract

**Background:**

Successful implementation and sustainment of interventions is heavily influenced by context. Yet the complexity and dynamic nature of context make it challenging to connect and translate findings across implementation efforts. Existing methods to assess context are typically qualitative, limiting potential replicability and utility. Existing quantitative measures and the siloed nature of implementation efforts limit possibilities for data pooling and harmonization. The Inventory of Factors Affecting Successful Implementation and Sustainment (IFASIS) was developed to be a pragmatic, quantitative, organizational-level assessment of contextual factors. The intention is to characterize context with a measure that may enhance replication and reproducibility of findings beyond single implementation case studies. Here, we present the development and validation of the IFASIS.

**Methods:**

A literature review was conducted to identify major concepts of established theories and frameworks to be retained. IFASIS data were examined in relation to implementation outcomes gathered from two studies. Psychometric validation efforts included content and face validity, reliability, internal consistency, and predictive and concurrent validity. Predictive validity was evaluated using generalized estimating equations (GEE) for longitudinal data on three implementation outcomes: reach, effectiveness, and implementation quality. Pragmatic properties were also evaluated.

**Results:**

The IFASIS is a 27-item, team-based, instrument that quantitatively operationalizes context. Two rating scales capture current state and importance of each item to an organization. It demonstrated strong reliability, internal consistency, and predictive and concurrent validity. There were significant associations between higher IFASIS scores and improved implementation outcomes. A one-unit increase in total IFASIS score corresponded to a 160% increase in the number of patients receiving a medication (reach). IFASIS domains of factors outside the organization, factors within the organization, and factors about the intervention, and subscales of organizational readiness, community support, and recipient needs and values, were predictive of successful implementation outcomes. IFASIS scores were also significantly associated with measures of implementation quality.

**Conclusions:**

The IFASIS is a psychometrically and pragmatically valid instrument to assess contextual factors in implementation endeavors. Its ability to predict key implementation outcomes and facilitate data pooling across projects suggests it can play an important role in advancing the field.

Contributions to the literature

- The IFASIS is a quantitative measure designed to characterize any context and any innovation endeavor.
- The IFASIS has acceptable psychometric properties and pragmatic utility.
- The IFASIS may be useful for implementation research and practice.
- The IFASIS allows for pooling data across projects that could accelerate generalizable findings.

## Background

The public health impact of implementation research is shackled by variation in approach and method, rendering cross-study comparability, reproducibility, and pooling data for meta-analyses impossible [1–5]. This is particularly evident in methods commonly used to examine context. Within determinant frameworks, contextual factors can make or break an implementation endeavor [6, 7].

Contextual factors, such as leadership culture, policies, and financing, can influence the process and outcomes of implementation efforts [6]. They should also drive the adaptation of the innovation being implemented and inform the selection and tailoring of implementation strategies [8, 9]. Yet, no two contexts are alike, nor are they static. Context is complex, multilayered, and dynamic [10].

Given this complexity, as well as the seemingly unique nature of context in any implementation activity, implementation science tends to rely on qualitative methods to explore important contextual factors. Qualitative studies, though informative and rich, suffer from a lack of replicability. They are also time-consuming, and not well suited to rapidly evaluate changes in context over time, throughout an implementation endeavor, or across projects. Though rigorous, rapid methods to collect and analyze qualitative data exist, they do not provide instant or close to instant feedback to organizations engaged in implementation efforts or research teams [11–14]. Obtaining this type of information in a time-sensitive manner is often necessary for decision making at an organizational level. Furthermore, while rapid qualitative methods are much faster than traditional approaches, they are not within reach in non-research settings where teams do not have the bandwidth or skill set for data collection and analyses.

Current quantitative methods to evaluate context tend to focus on a specific aspect of context, such as leadership, organizational readiness, or a particular setting (e.g., schools, hospitals) [15–17]. Though there is tremendous value in being able to dive deep on a specific aspect of context, being able to assess breadth of context in a pragmatic manner is necessary [6]. Frameworks such as the Consolidated Framework for Implementation Research (CFIR) do cover multiple elements of context but lend themselves better to qualitative inquiries [18–20]. The existing instruments available that evaluate single constructs of context use different rating scales, not allowing for the pooling of data across projects [21]. They also tend to rely on individual surveys.

To best serve partners in implementation efforts who have finite resources, and to move towards closing the research-to-practice gap, the field needs methods to characterize context that balance rigor and pragmatism and to do so efficiently given often strained resources [22]. The siloed conduct of implementation projects compounded with the lack of consistency by which to evaluate context, and the ability to pool data across projects, further impedes the field’s ability to generate generalizable findings, methods, and tools.

To address these limitations, and to fulfill the public health and scientific promise of implementation research, we aimed to develop and evaluate a quantitative measure of key contextual factors that drive the implementation process.

The purpose of this article is to present the development and validation of the Inventory of Factors Affecting Successful Implementation and Sustainment (IFASIS), as well as its applicability to evaluate context and generalize findings. We report on its psychometric and pragmatic evidence following the Psychometric and Pragmatic Evidence Rating Scale [29] and COnsensus-based Standards for the selection of health status Measurement INstruments (COSMIN) guidelines [30].

## Methods

### Measure development

The development of the IFASIS was driven by four priorities to maximize its usefulness and impact [29, 31, 32]. It needed to a) be able to characterize any context or innovation endeavor, b) have acceptable pragmatic and psychometric properties, [29, 31] c) have practical utility, and d) allow for pooling data across projects to accelerate generalizable findings. There was also an intent to develop a measure that would follow a similar structure and methodology as other widely used, validated instruments [33–43] previously developed by our team.

A comprehensive literature review was conducted to determine guiding principles and key constructs to be included. This allowed for the examination and synthesis of existing work in this space, with a focus on identifying: 1) current gaps in the evaluation of contextual determinants, and 2) predominant theories, frameworks, and constructs pertaining to contextual determinants [44]. Examples of terms searched in PubMed and Google Scholars are: context, barriers, facilitators, determinants, equity, implementation. The “Assess” section of the Dissemination and Implementation Models in Health website [45] about barriers and facilitators was thoroughly reviewed [46]. Most cited works identified via this website were added to the literature review. Once guiding principles and key constructs were identified, a conceptual model was developed. The conceptual model, presented in the Results section, framed the development of the items to be included in, and format of, instrument.

### Validation

Because the IFASIS is meant to balance psychometric and pragmatic properties to meet the needs of implementation efforts, its validation was guided by PAPERS (the Psychometric and Pragmatic Evidence Rating Scale) and COSMIN (COnsensus-based Standards for the selection of health status Measurement INstruments) [29–31].

#### Content validation

Content validity is the degree to which the content of an instrument accurately reflects the constructs to be measured. It is usually paired with face validity, the degree to which items of an instrument reflect the constructs to be measured. Following an iterative content and process field testing, user (researcher and non-researcher) feedback was solicited. The IFASIS was perceived to be relatively easy to use and informative, with a clear purpose. Recommendations were made to simplify language of some items and ratings. The next step in measure refinement was a thorough review by seven faculty from the Research Core of the National Institute on Drug Abuse–funded P50 Center of Excellence: the Center for Dissemination and Implementation At Stanford (C-DIAS). This group of implementation research experts are established investigators with experience in measure development; theories, models, and frameworks; qualitative research and ethnography; matching implementation strategies to contextual factors; and health equity. This group verified the face validity and the potential usefulness and versatility of the IFASIS. They recommended a reduction in the number of items, and being clear about data collection methods, target sample and audiences, and data yield, summarization, and interpretation. Feedback from both the field testing and expert reviews was incorporated and a revised, refined version of the IFASIS was generated. The final version of the IFASIS was deployed in three implementation projects. Longitudinal data from two of these three projects serve as the study sample used to evaluate reliability, internal consistency, and predictive validity [29–31].

### Study sample

The Addiction Treatment Starts Here (ATSH) and Stagewise Implementation-To-Target Medications for Addiction Treatment (SITT-MAT) [52, 53].

ATSH is a project to increase access to medications for addiction treatment in safety-net primary care clinics across California [52]. Inclusion criteria for clinics were: 1) providing care within the State of California, 2) providing comprehensive primary care services to underserved populations, 3) meeting the definition of a non-profit and tax-exempt entity under 501(C) [3] of the Internal Revenue Service Code or a governmental, tribal, or public entity. This includes Federally Qualified Health Centers (FQHCs) and FQHC look-alikes, community clinics, rural health clinics, free clinics, ambulatory care clinics owned and operated by public hospitals, and Indian Health Services clinics; and 4) were not “new” to medications for opioid use disorder (MOUD). This was defined as having an established MOUD program that has been in operation for not less than one year, with multiple active prescribers working within an established multidisciplinary team that meets regularly to maintain and improve the MOUD program. Since its launch in 2019, ATSH has supported 97 safety-net clinics in designing new or expanding existing MOUD programs across four waves. Implementation strategies used to that end include 1) Enhanced Monitoring and Feedback, 2) Learning Collaboratives, 3) External Facilitation, and 4) Didactic Webinars. Clinics in Wave 4, n = 20, completed the IFASIS at midpoint and endpoint of the implementation process. Implementation outcomes for program evaluation are reach, adoption, and implementation quality.

SITT-MAT is a five-year, NIDA-funded R01 designed to improve patient access to medications for opioid use disorder within specialty addiction programs and primary care clinics in Washington State [53]. Inclusion criteria for organization include: 1) providing care in Washington State, and 2) identifying as one of the following clinic types: addiction treatment programs, either residential (detoxification or rehabilitation) or outpatient (intensive outpatient or outpatient) levels of care, or primary care clinics, including FQHCs and Community Health Centers (CHCs). Opioid treatment programs were excluded from participation.

SITT-MAT partners with the Washington State Health Care Authority to offer implementation support to 24 primary care clinics and 46 specialty addiction treatment programs based on target implementation outcomes using a stepped implementation model where implementation supports of increasing intensity and cost are delivered only if less intensive implementation support do not achieve target outcomes. Implementation support strategies are enhanced monitoring and feedback (EMF), two-day NIATx workshop, internal facilitation, and external facilitation. Target implementation outcomes are achieving 75% reach, implementation capability above average, and having an integrated prescriber. Clinics were asked to complete the IFASIS after each implementation strategy; eight clinics completed the IFASIS post-EMF by the time of this analysis.

The final sample comprised 84 observations from 20 ATSH clinics and eight SITT-MAT clinics.

### Variables

In addition to administering the IFASIS at multiple time points, both studies collected data to evaluate RE-AIM implementation outcomes of reach, effectiveness, and implementation quality [54, 55]. In both ATSH and SITT-MAT, RE-AIM variables of reach, effectiveness, and implementation represent performance.

### IFASIS

IFASIS total score, domain scores, and subscale scores were derived by averaging ratings.

#### Reach

Each clinic contributed three repeated measures for the reach outcome. The two outcomes of reach were: a) the number of all patients prescribed MOUD, which is operationalized as the total number of patients administered MOUD in the past month. It includes patients who may be new, restarted, or established, and b) the number of new patients, diagnosed with opioid use disorder, who were prescribed and started MOUD within 30 days of diagnosis. Both operationalization of reach include patients who restarted MOUD after a 60-day or more break in treatment.

#### Effectiveness

Each clinic contributed two repeated measures for effectiveness. Effectiveness was operationalized as the percent of new patients engaged in MOUD, meaning of the total number of patients prescribed MOUD in the past month, the proportion of patients who had two or more in-person outpatient clinical visits within 34 days of starting MOUD [56].

#### Implementation quality

Implementation quality was operationalized using the total Integrating Medications for Addiction Treatment (IMAT) score. Scoring for this instrument has been described by Chokron Garneau et al. [40] IMAT items are rated on a 5-point scale ranging from 1-Not Integrated, 3-Partially Integrated, to 5-Fully Integrated. The total score is derived by averaging all items and yields a composite rating from 1 to 5 of overall implementation quality. A higher score reflects better implementation quality.

### Statistical analyses

#### Reliability

Reliability evaluates the extent to which a measure is free from measurement error [57]. Reliability over time was evaluated by calculating the intraclass correlation coefficient (ICC) for IFASIS scores across time 1 and time 2, using a two-way random effects model for the mean response. Clinics that had complete data for both time points were included, resulting in a reduced dataset of *n* = 21 observations for this analysis.

#### Internal consistency

To assess internal consistency, the degree of interrelatedness among the items, we calculated Chronbach’s alpha for the IFASIS responses at each time point.

#### Predictive validity

To evaluate the predictive validity of the IFASIS on outcomes of reach, generalized estimating equations (GEE) approach for longitudinal data was used. The GEE approach is a useful approach for longitudinal data when the response variable is discrete (e.g., ordinal or count) [58]. When the response outcome is discrete, linear mixed models are not appropriate. GEE allows for flexible modeling with repeated measures over time and for the specification of different distributions given that the variables of interest are counts. In longitudinal studies where outcomes for participants are observed multiple times, responses are expected to be correlated. GEEs account for this within-participant correlation. In addition, GEEs estimate population-averaged effects, which are useful when the interest is in understanding the average response in the population rather than participant-specific effects.

Summary statistics and data visualization techniques were used to describe the data distribution. We specified the family of the response variable as negative binomial and assumed an exchangeable correlation structure. Twenty-eight clinics with 84 observations were used for this analysis. Exponentiated coefficients, which can be interpreted as risk ratios (RRs), are reported to facilitate interpretation, as estimated coefficients are on the log-scale. The RR represents the change in the outcome for each one-unit increase in the IFASIS score, where RR > 1 represents an increase, RR < 1 represents a decrease, and RR = 1 represents no change in the outcome. Total IFASIS score, domains, and subscales were examined for predictive validity.

To evaluate predictive validity on effectiveness, GEE was used with a Gaussian normal distribution. Twenty-five clinics with 55 observations were retained for this analysis given missing data. For all analyses, baseline IFASIS scores and clinic type (primary versus specialty care) were accounted for. It was hypothesized that higher IFASIS scores (lower barriers) would be associated with higher reach and effectiveness. Furthermore, primary care clinics were expected to outperform specialty care clinics as MOUD care tends to be better established in the latter [53, 59].

#### Concurrent validity

Concurrent validity was assessed by examining the relationship between the IFASIS total score and implementation quality using a linear model. Significant barriers should be associated with lower implementation quality.

## Results

### Conceptual model

**Fig. 1:**
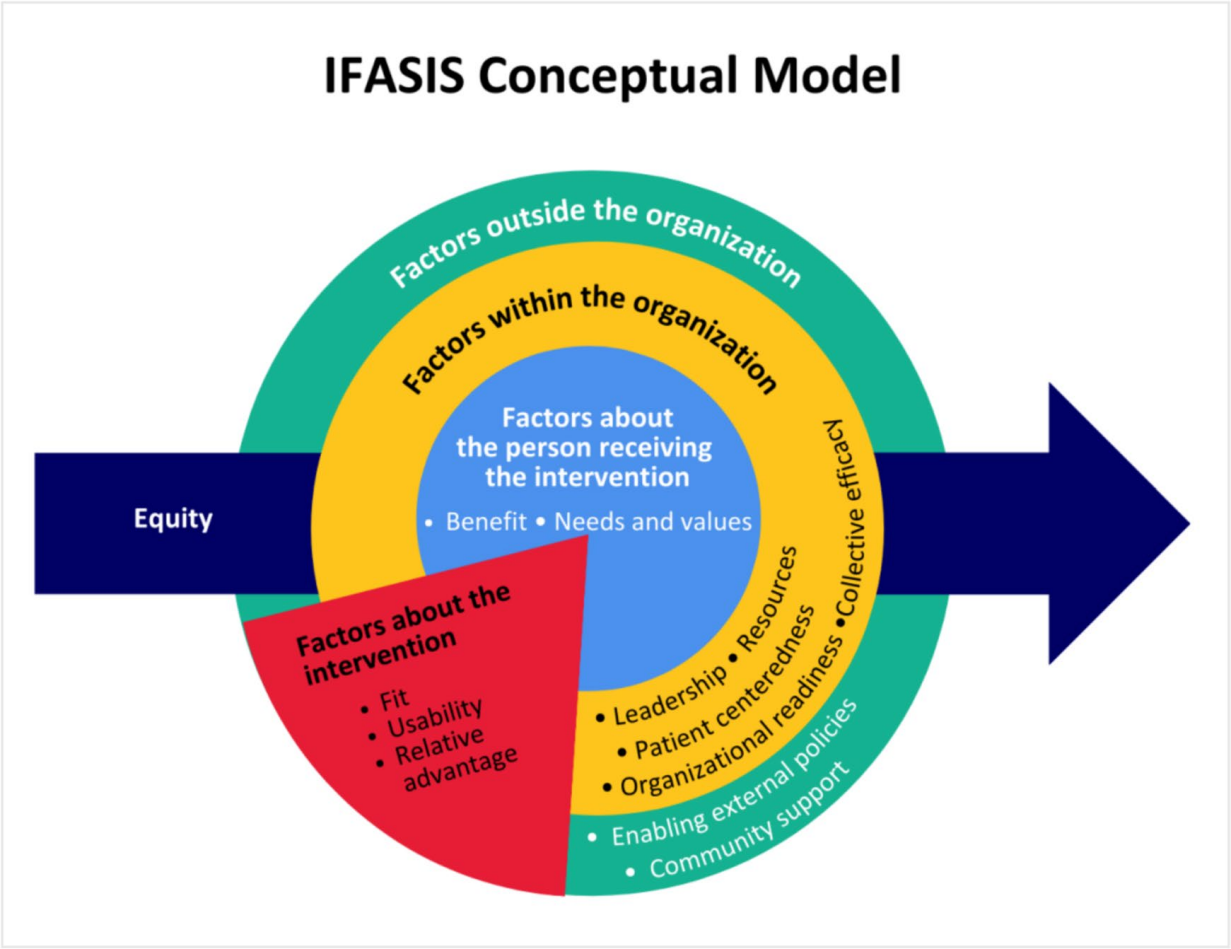
IFASIS Conceptual Model

The IFASIS’ conceptual model is illustrated in Fig. 1. Guiding principles of the model are that context is conceptualized as multileveled and dynamic; equity should be considered across all domains and not solely at the recipient level; and determinants are interrelated, including those of the intervention.

Drawing from the CFIR; Exploration, Preparation, Implementation, Sustainment (EPIS); Normalization Process Theory (NPT); Health Equity Implementation Framework (HEIF); Organization Theory; and work by Mendel et al. and Squires et al. [15, 18–20, 23, 24, 27, 28, 47–51], 13 constructs were retained for inclusion in the measure. These constructs were then organized into four domains: factors outside the organization, factors within the organization, factors about the intervention, and factors about the person receiving the intervention. Twenty-seven items were generated to operationalize the 13 constructs (subscales). Definitions of domains, constructs (subscales), and their originating theories and frameworks are presented in Table 1. Constructs included in the IFASIS are by no means exhaustive or meant to suggest that these are the constructs that should matter the most to all organizations. They are simply a starting point, gathering most referred to and used constructs to date.

**Table 1:** Domains, Subscales of the IFASIS and their Originating Theory or Framework

Domains	Constructs (Subscales)	Source
Factors Outside Your Organization	External Policies, Community Support	EPIS, CFIR, Organizational Theory, i-PARIHS
Factors Within Your Organization	Leadership, Resources, Organizational Readiness, Do-ability, Person Focused Care	EPIS, CFIR, Organizational Theory, HEIF
Factors About the Intervention	Fit, Usability/Complexity, Relative Advantage	EPIS, CFIR, Organizational Theory, HEIF, i-PARIHS
Factors About the Person Receiving the Intervention	Benefit to Recipient, Recipient Needs and Values	HEIF, i-PARIHS

The Health Equity construct is derived from 7 items that cut across all domains and subscales. Items for the Health Equity subscale are informed by the HEIF and i-PARIHS

### Scoring

A scoring methodology similar to that of other widely used and validated instruments [33–43] previously developed by our team was retained. Each of the 27 items is scored on two scales. The first scale, referred to as rating, ranges from 1 to 5, and reflects the status of a given item within an organization. It is modeled on rating scales used in widely used, validated instruments [33–43]. For example, ratings for the item “Staff shortage and turnover within an organization” are 1) A serious issue: there is a lack of qualified staff to deliver [INTERVENTION], which poses significant challenges; 2) Between 1 and 2; 3) Challenging but manageable: while there are some staff members with experience, high turnover rates remain a concern; 4) Between 3 and 5; and 5) Not an issue for our organization; we have enough qualified staff to deliver [INTERVENTION]. Ratings of 2 and 4 are indicated for in-between circumstances. Organizations are encouraged to select 2 or 4 when they do not fully meet the next-level anchors of 3 and 5. Sample IFASIS items and their respective rating scales are presented in Table 2.

**Table 2:** Sample IFASIS items and rating scale

Domains & Subscales	Items	1	2	3	4	5
**Factors outside your organization**						
Community Support	*Consultations with community members about the overall fit and acceptability of [INTERVENTION] from a diversity and inclusivity perspective are…*	Non-existent; no consultations with community members	Between 1&3	Mixed; occasional consultations with community members	Between 3&5	Ongoing and frequent; routine consultations with community members
**Factors within your organization**						
Person Focused Care	*Our program collects and examines patient data by demographic or other social indicators to identify potential inequities in delivery of [INTERVENTION]…*	Program collects demographic data but does not examine it for potential inequities	Between 1&3	Program collects demographic data and examines it for potential inequities, but the results are not shared within the clinic	Between 3&5	Program collects demographic data for potential inequities & uses it to set quality improvement aims to address inequity, sharing results within the clinic regularly
**Factors about the intervention**						
Fit	*[INTERVENTION]* *fit for our organization and patients…*	Most patient care staff are not convinced that this intervention is a suitable fit for our organization and patients	Between 1&3	Patient care staff hold mixed opinions, with some believing that intervention is a good fit for patients while others have reservations	Between 3&5	Patient care staff are firmly convinced that this intervention is a suitable fit for both our organization and patients
**Factors about the persons receiving the intervention**						
Recipients Needs and Values	*Adaptability to patient’s* *cultural beliefs…*	[INTERVENTION] is not adaptable to patients’ cultural beliefs. It cannot be tailored and delivered in a way that fits patients’ cultural beliefs	Between 1&3	[INTERVENTION] is somewhat adaptable to patients’ cultural beliefs. Some aspects can be tailored and delivered to fit patients’ cultural beliefs	Between 3&5	[INTERVENTION] is very adaptable to patients’ cultural beliefs, it can be tailored and delivered in a way that fits patients’ cultural beliefs

The second scale, importance, reflects the importance a team attributes toward an implementation effort. Importance is rated on a 3-point scale: Not important, Somewhat important, Important. This dual scale allows teams to reflect on the importance attributed to each factor and whether an item is a barrier, opportunity, or advantage to an implementation effort. Total IFASIS score ranges from 27 to 135 in terms of ratings, and 27 to 81 for the importance scale.

Based on prior success with team-based, organizational assessments, similar instructions for the data collection and completion of the IFASIS were retained [33–43]. Organizations aiming to use the IFASIS are instructed to assemble a team of three to seven people with various ranks and roles in the organization who will be using the innovation in a research study, implementation endeavor, or quality improvement project. Teams review the IFASIS items one by one and select the rating that reflect the current status in their organization and the importance they attribute to each item. Consensus needs to be reached for scoring. Ratings are not meant to be overly difficult to choose, and respondents are encouraged to select the lower of two ratings if unsure. The IFASIS takes between 30 and 45 min to complete.

In addition to providing a balance between individual self-report and external observation, bringing team members together to complete the IFASIS provides an opportunity to discuss each item and its status and importance. This method focuses on the review and discussion of each item content at a time in contrast to key informant group interviews where multiple themes can emerge for a given prompt.

Descriptive statistics of clinic characteristics are presented in Table 3.

**Table 3:** Baseline clinic characteristics (*N* = 28)

Clinic Characteristics	*N*	%
Clinic type		
Primary care	21	75.0
Specialty care	7	25.0
Rurality		
Urban/metropolitan	25	89.3
Rural	3	10.7
Medically underserved		
Yes	10	35.7
No	18	64.3
Mental health provider shortages		
Yes	16	57.1
No	12	42.9
	**Mean**	**SD**
Staffing		
Physicians	8	15.8
Nurse practitioners or physician assistants	4	8.3
Registered nurses or licensed practical nurses	3	5.0
Behavioral health or mental health clinicians	3	3.4
Clinic managers or clinic supervisors	2	4.5
Insurance type		
Patients on Medicaid (%)	69.5	22.1
Patients on Medicare (%)	11.0	10.2
Patients with dual eligibility (%)	5.5	9.1
Patients on private insurance (%)	5.4	7.2
Uninsured patients (%)	8.8	7.2

Most clinics were primary care clinics (21, 75.0%) in urban or metropolitan areas (25, 89.3%). Clinics served communities that are medically underserved (10, 35.7%) and experience mental health provider shortages (16, 57.1%), with most patients on Medicaid (69.5%), on Medicare (11.0%), or uninsured (8.8%). Median total scores and interquartile ranges for the IFASIS at both time points are presented in Table 4. Median total scores and interquartile ranges for IMAT were 3.64 (3.36–4.05) and 4.10 (3.75–4.42), respectively.

**Table 4:** Descriptive statistics of IFASIS

IFASIS Scores	T1 (*n* = 28) Mean (SD)	Median [IQR]	T2 (*n* = 16) Mean (SD)	Median [IQR]
**Total**	3.95 (0.49)	4.06 [3.58–4.31]	4.17 (0.50)	4.17 [3.89–4.56]
**Domains**				
Outside your organization	3.44 (0.76)	3.40 [2.95–4.00]	3.95 (0.68)	3.80 [3.55–4.50]
Within your organization	3.97 (0.55)	4.04 [3.73–4.25]	4.09 (0.53)	4.21 [3.88–4.42]
About the intervention	4.38 (0.56)	4.33 [4.00–5.00]	4.56 (0.50)	4.67 [4.33–5.00]
About person receiving intervention	4.08 (0.50)	4.14 [3.82–4.32]	4.30 (0.52)	4.43 [4.00–4.71]
**Subscales**				
External Policies	3.77 (0.88)	3.75 [3.00–4.50]	4.38 (0.74)	4.75 [4.00–5.00]
Community Support	3.23 (0.87)	3.00 [2.67–3.75]	3.67 (0.81)	3.50 [3.00–4.42]
Leadership	3.95 (0.87)	4.00 [3.50–4.63]	4.00 (0.88)	4.00 [3.50–4.63]
Resources	3.82 (0.77)	4.00 [3.58–4.33]	3.92 (0.63)	4.00 [3.58–4.33]
Organizational Readiness	4.18 (0.69)	4.33 [3.92–4.67]	4.35 (0.49)	4.33 [4.00–4.75]
Doability	4.29 (0.81)	4.50 [4.00–5.00]	4.19 (1.05)	5.00 [3.00–5.00]
Person Focused Care	3.82 (0.72)	3.83 [3.33–4.42]	4.02 (0.76)	4.00 [3.67–4.67]
Fit	4.39 (0.69)	4.50 [4.00–5.00]	4.56 (0.73)	5.00 [4.00–5.00]
Usability/Complexity	4.36 (0.62)	4.00 [4.00–5.00]	4.50 (0.73)	5.00 [4.00–5.00]
Relative Advantage	4.39 (0.69)	4.50 [4.00–5.00]	4.63 (0.62)	5.00 [4.00–5.00]
Benefit to Recipient	4.79 (0.50)	5.00 [5.00–5.00]	4.88 (0.50)	5.00 [5.00–5.00]
Recipient Needs and Values	3.96 (0.56)	4.00 [3.67–4.33]	4.21 (0.59)	4.33 [3.83–4.67]
Health Equity	3.89 (0.51)	3.86 [3.54–4.29]	4.12 (0.56)	4.21 [3.57–4.46]

### Internal consistency

Chronbach’s alpha was 0.94 at time 1 and 0.97 at time 2, respectively, indicating excellent internal consistency [29, 31].

### Reliability

The intraclass correlation coefficient was 0.966 (95% CI [0.941, 0.984]), indicating strong reliability.

### Predictive validity

#### Reach

Total IFASIS score was significantly associated with the number of new and established patients, increasing by 160% for every unit increase in the total IFASIS score (RR = 2.60, *p* = 0.01). Three of four IFASIS domains significantly predicted the number of patients, where a one-unit increase in the “factors outside your organization” domain corresponded to an 82% increase of patients on medications (1.8, *p* = 0.09), while the “factors within your organization” and “factors about the intervention” domains each corresponded to a 110% increase in patients (RR = 2.1, *p* ≤ 0.05). Several IFASIS subscales were also significantly associated with the number of patients on medication (*p* ≤ 0.05), including Recipients Needs and Values (RR = 2.2), Organizational Readiness (RR = 2.1), Fit (RR = 2.1), and Community Support (RR = 1.9). Across all models, there was a difference between primary and specialty care clinics (range of RR = [3.6, 4.6], *p* < 0.05) as expected.

Total IFASIS and domains scores did not significantly predict the number of new patients on medication. However, subscales of Community Support (RR = 1.7), and Organizational Readiness (RR = 1.8) were predictive (*p* ≤ 0.05). Across all models there was a difference between primary and specialty care clinics.

#### Effectiveness

Regarding effectiveness, only the domain of “factors about the intervention”, and the subscale of Usability/Complexity were significant. A unit increase in these domain scores were associated with a 14.8% and 19% increase in retention, respectively (p ≤ 0.05).

#### Concurrent validity

IMAT (Implementation Quality) Total score was significantly associated with IFASIS Total score (β = 0.47, *p* = 0.01).

Tables 5 and 6 present Risk Ratios of IFASIS Domains on MOUD Patients.

**Table 5:** Risk Ratios of IFASIS Domains on New and Existing MOUD Patients: Results from Generalized Estimating Equations (GEE)

Model	Risk Ratio	Standard Error	*p* Value
Intercept	0.181	1.953	.234
Total IFASIS	2.598	0.481	.011
Time	0.988	0.026	.613
Primary care clinic	7.698	0.547	.000
Intercept	0.954	1.332	.969
Factors outside your organization	1.819	0.352	.089
Time	0.998	0.025	.921
Primary care clinic	8.099	0.619	.000
Intercept	0.447	1.778	.595
Factors within your organization	2.122	0.437	.051
Time	0.991	0.020	.662
Primary care clinic	6.747	0.553	.000
Intercept	0.346	1.782	.424
Factors About the Intervention	2.113	0.405	.013
Time	0.978	0.029	.389
Primary care clinic	6.578	0.535	.000
Intercept	0.464	2.125	.630
Factors about the persons receiving the intervention	2.013	0.495	.070
Time	0.982	0.024	.405
Primary care clinic	7.990	0.579	.000
Intercept	9.570	1.212	.044
External policies	1.007	0.310	.984
Time	0.986	0.020	.459
Primary care clinic	6.649	0.626	.002
Intercept	0.840	1.161	.868
Community support	1.889	0.305	.037
Time	0.997	0.029	.914
Primary care clinic	8.675	0.620	.000
Intercept	1.604	1.197	.691
Leadership	1.512	0.287	.200
Time	0.995	0.024	.840
Primary care clinic	7.526	0.572	.000
Intercept	1.946	1.263	.598
Resources	1.638	0.331	.084
Time	0.989	0.018	.543
Primary care clinic	4.304	0.584	.006
Intercept	0.292	1.612	.255
Organizational readiness	2.132	0.358	.003
Time	0.993	0.022	.739
Primary care clinic	9.327	0.561	.000
Intercept	0.582	1.304	.694
Doability	1.807	0.282	.063
Time	0.980	0.023	.348
Primary care clinic	8.476	0.532	.000
Intercept	4.418	1.499	.386
Person-focused care	1.236	0.352	.585
Time	0.988	0.019	.501
Primary care clinic	6.425	0.592	.002
Intercept	0.433	1.514	.516
Fit	2.055	0.335	.004
Time	0.972	0.029	.256
Primary care clinic	5.530	0.533	.002
Intercept	0.212	1.602	.224
Usability	2.251	0.356	.008
Time	0.985	0.031	.581
Primary care clinic	8.425	0.521	.000
Intercept	5.230	1.669	.244
Relative advantage	1.156	0.382	.661
Time	0.985	0.020	.444
Primary care clinic	6.545	0.611	.001
Intercept	240.175	2.407	.001
Benefit to recipient	0.521	0.500	.075
Time	0.985	0.021	.491
Primary care clinic	5.344	0.580	.004
Intercept	0.331	1.748	.478
Recipient needs and values	2.211	0.412	.036
Time	0.979	0.027	.380
Primary care clinic	8.114	0.539	.000

**Table 6:** Risk Ratios of IFASIS Domains on New MOUD Patients: Results from Generalized Estimating Equations (GEE)

Model	Risk Ratio	Standard Error	*p* Value
Intercept	0.444	1.657	.505
Total IFASIS	1.646	0.407	.133
Time	1.011	0.052	.823
Primary care clinic	4.040	0.475	.012
Intercept	0.632	1.049	.629
Factors outside your organization	1.586	0.273	.084
Time	1.002	0.056	.972
Primary care clinic	4.052	0.488	.011
Intercept	0.795	1.522	.850
Factors within your organization	1.434	0.373	.256
Time	1.016	0.051	.747
Primary care clinic	3.830	0.485	.019
Intercept	2.448	1.589	.344
Factors about the intervention	1.084	0.361	.680
Time	1.012	0.051	.800
Primary care clinic	3.702	0.490	.025
Intercept	0.542	1.652	.620
Factors about the persons receiving the intervention	1.526	0.383	.199
Time	1.015	0.052	.759
Primary care clinic	4.277	0.462	.005
Intercept	2.742	0.947	.188
External policies	1.064	0.240	.763
Time	1.014	0.051	.767
Primary care clinic	3.713	0.492	.029
Intercept	0.528	0.921	.415
Community support	1.678	0.237	.014
Time	0.990	0.059	.872
Primary care clinic	4.417	0.493	.005
Intercept	1.236	0.983	.809
Leadership	1.272	0.234	.340
Time	1.012	0.054	.820
Primary care clinic	4.029	0.473	.013
Intercept	2.277	1.047	.313
Resources	1.130	0.273	.527
Time	1.012	0.051	.810
Primary care clinic	3.484	0.496	.034
Intercept	0.281	1.410	.271
Organizational readiness	1.726	0.311	.055
Time	1.014	0.050	.761
Primary care clinic	4.788	0.495	.002
Intercept	0.578	1.173	.593
Doability	1.467	0.251	.118
Time	1.012	0.050	.818
Primary care clinic	4.293	0.487	.007
Intercept	3.957	1.206	.322
Person-focused care	0.967	0.282	.901
Time	1.013	0.052	.794
Primary care clinic	3.714	0.490	.030
Intercept	1.542	1.323	.710
Fit	1.213	0.292	.343
Time	1.008	0.052	.870
Primary care clinic	3.516	0.478	.030
Intercept	1.059	1.447	.957
Usability	1.291	0.322	.362
Time	1.002	0.052	.962
Primary care clinic	4.101	0.482	.013
Intercept	10.765	1.290	.023
Relative advantage	0.765	0.295	.196
Time	1.014	0.053	.765
Primary care clinic	3.860	0.486	.016
Intercept	8.958	2.004	.044
Benefit to recipient	0.823	0.415	.449
Time	1.012	0.051	.813
Primary care clinic	3.624	0.497	.032
Intercept	0.541	1.455	.561
Recipient needs and values	1.537	0.341	.133
Time	1.014	0.052	.775
Primary care clinic	4.374	0.461	.004

### Practical utility

#### Visualization and data feedback to organizations

**Fig. 2:**
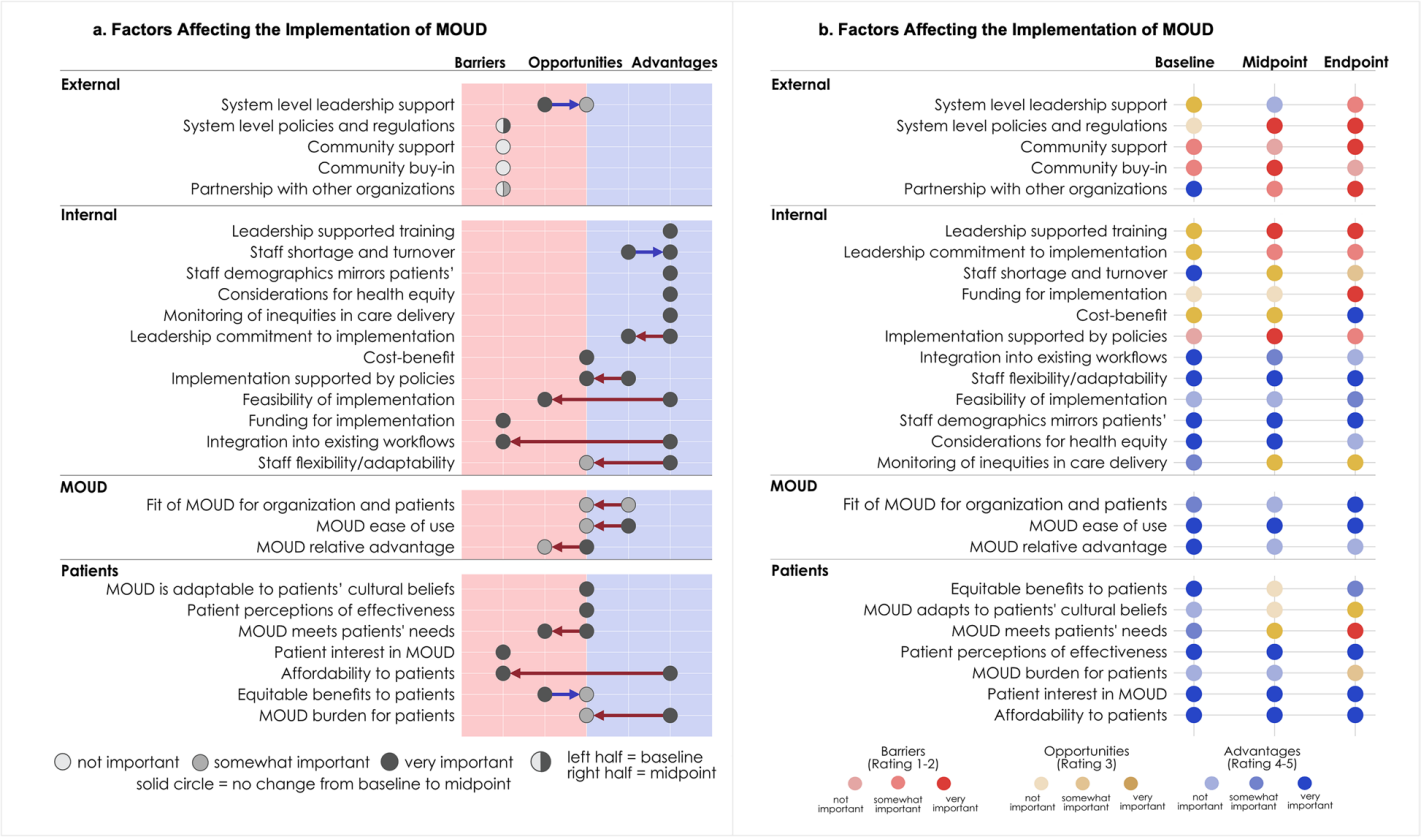
IFASIS Visualization, Scores at Pre and Active Implementation. Figure 2a Legend: The figure illustrates the barriers, things that get in the way, and facilitators, things that help, with the implementation of medications for opioid use disorder (MOUD) programs as identified by a team at two different time points: at baseline and after 6 months. The color of the circles (light gray – not important, medium gray – somewhat important, dark gray – very important) reflects the importance a team attributed to each factor. In addition to the importance levels, the figure includes directional arrows capturing the change in each factor over the six-month period. An arrow points from the position of the factor at baseline toward its position at the six-month mark. For example, “Patient Interest in MOUD” went from being a very important barrier at baseline to being a somewhat important facilitator after 6 months that a team can continue to leverage and build on to implement MOUD programs. Figure 2b Legend: The figure illustrates the barriers, opportunities, and advantages associated with implementing MOUD programs as identified by a team at three time points: baseline, midpoint, and endpoint. The x-axis represents time (baseline, midpoint, and endpoint), while the y-axis lists factors that can influence implementation. Each factor is categorized as a barrier (red), opportunity (yellow), or advantage (blue), with its importance indicated by color opacity: lighter shades represent less important factors, while darker shades indicate more important ones. A legend at the bottom of the figure clarifies these color and opacity distinctions. For example, “Cost–benefit” was a very important opportunity (dark yellow) at both baseline and midpoint. At the endpoint, it had shifted to being a very important facilitator (dark blue), reflecting a change this factor over time

The IFASIS was also designed to be a useful tool to feed information back to organizations and stimulate discourse with implementation participants and partners about barriers and facilitators. In addition to quantitative data being fed back to teams, an accompanying visualization (Fig. 2a and b) illustrates barriers, facilitators, and opportunities at a glance, their importance, and change over time. The IFASIS visuals have successfully been used to inform conversations with partners for implementation strategy selection and planning [60].

## Discussion

### Main findings

The Inventory of Factors Affecting Successful Implementation and Sustainment (IFASIS) was developed to address an existing gap pertaining to the assessment of context – the need for a pragmatic, quantitative, organizational-level assessment of contextual factors. The intention was to be able to characterize context with a measure that included key concepts of major theories and frameworks, and enhanced replication and reproducibility of findings beyond single implementation case studies.

Thirteen constructs from major theories and frameworks were retained and organized along four dimensions reflecting outer context, inner context, the interventions, and the recipients of the intervention. Each of the 27 items is scored based on status within the organization and importance to the implementation effort.

Validation efforts determined the IFASIS to be a valid, pragmatically useful instrument to evaluate barriers and facilitators to an implementation process and their changes over time. IFASIS ratings significantly predicted increases in implementation outcomes of reach and effectiveness and were associated with implementation quality. It also has strong reliability and internal consistency, on par with or superior to other well-established measures of determinants [16, 17, 61, 62]. The IFASIS fills a unique and critical gap in assessing determinants of implementation success or failure.

### Comparison with other contextual factor measurements

Compared to other available tools that serve to evaluate specific contextual determinants, the IFASIS: leverages a team-based approach, does not focus on a single aspect of context, is quantitative and allows for pooling of data, can be used as a repeated measure to capture change over time, considers the importance of each item to an organization, and can be used in the selection and planning of implementation strategies.

Teams are asked to review and discuss each IFASIS item to reach consensus scoring. Contrary to individual measures, this provides teams with an opportunity to share potentially differing perspectives allowing them to balance ratings while working together to establish priorities. [33–43] A team-based approach to scoring also addresses issues of reliability encountered when aggregating individual quantitative measures [63, 64]. Participants are encouraged to select the lower of two ratings if they are in between because in organizational settings the higher the rating, the harder it will be to demonstrate improvement over time. It is meant to curb positive response bias in self-assessment of organizations. This recommendation is made in all the organizational capability measures developed by our team [33–43].

Validation efforts also demonstrated that data can be pooled across projects, allowing for reproducibility, comparability, and greater transparency and impact. Here, we were able to combine data from multiple sites, across two research projects to evaluate its psychometric and pragmatic properties. Regarding pragmatic properties, the IFASIS meets the “excellent” criteria for cost, language, ease of training, and interpretation, and the “good” rating for length per PAPERS [29, 31]. IFASIS scores during the exploration or preparation stages of an implementation effort can also guide the selection and tailoring on implementation strategies [60, 65–67].

The IFASIS results and visualization also stimulate productive discourse with implementation participants and partners about barriers and facilitators, and the selection of implementation strategies. It has been used to elucidate both generalizable and context-specific implementation determinants, and to guide the provision of implementation facilitation [60]. Furthermore, the IFASIS and its visualization enable the evaluation of the interrelationship among determinants, and their change over time.

### Limitations

The IFASIS is by no means a comprehensive representation of all possible contextual determinants that may influence an implementation effort. Additionally, not all items or constructs included in the IFASIS are relevant to all implementation efforts. It is not meant to be prescriptive; it is a starting point. Furthermore, there is a tradeoff between the pragmatic nature of the IFASIS and the loss of richness and nuance that can be obtained from data gathered by qualitative interviews. Contextual determinants and their interrelationship may extend beyond what can be recorded by a quantitative instrument.

From an internal validity perspective, our sample size is small and both studies implemented the same innovation yet using different implementation strategies. The data used for this effort was collected in two different contexts (primary and specialty care clinics), additional diversity in types of organizations could have been beneficial. No data was recorded as to whom completed the IFASIS or team dynamics during the completion of the IFASIS. Although a team-based assessment has its advantages, some possible drawbacks include positive response bias, as well as power dynamics at play if leadership and/or supervisors are present during the exercise [68]. Another limitation of this study is that we did not perform factor analysis in order to validate the structural validity, due to the limited sample size.

### Implications and next steps

Next steps include continuing to improve the rigor and pertinence of the IFASIS. Forthcoming work includes conducting a confirmatory factor analysis (CFA) when more data are available. Because the IFASIS is intended for use by both researchers and non-researchers, there is value in conducting a CFA even though it may not indicate the relative importance of individual items within a subscale. For example, both community perception and policies are items are in the factors outside your organization domain. Though these two may not load on the same factor, ratings on both are meaningful to an organization planning an implementation project. From a researcher perspective it is however important to understand how well a set of items operationalizes a construct both from a measurement and an instrument refinement standpoint. Additional work to be done also includes comparing independent versus team-based assessment scoring, and including the consideration of the importance score. Finally, the IFASIS needs to be evaluated outside of the addiction health services space.

The IFASIS represents an important advancement in addressing the tension between context specificity and generalizability in implementation science. It does so by creating a common language for implementation researchers and practitioners that facilitates cross-context comparisons; identifying consistent determinant patterns across diverse settings while highlighting unique contextual profiles that require tailored approaches; and capturing key contextual factors in a quantifiable format for data aggregation across implementation efforts. The visualization component further enhances generalizability by providing a standardized method to communicate information across partner groups and settings. In summary, the IFASIS directly addresses implementation science's ongoing challenge of building cumulative knowledge while considering contextual uniqueness.

## Conclusions

The Inventory of Factors Affecting Successful Implementation and Sustainment is a quantitative, organizational-level team-based characterization of contextual factors and their relative importance. It draws from multiple well-established theories, models, and frameworks and integrates multilevel contextual constructs into a single 27-item instrument [18–20, 23–28]. It gathers information about factors that could influence efforts to implement a new intervention, program, or service, as well as their relative importance to an organization’s implementation efforts. It is highly adaptable to various innovations and settings and can be used to quantify barriers and facilitators, guide the selection and tailoring of implementation strategies, pool data across sites and projects, and provide immediate and useful feedback to implementation sites and teams. The IFASIS is currently being successfully used across multiple implementation projects with differing evidence-based practices being implemented and in different settings.

## Data Availability

Data can be made available upon request.

## Abbreviations

IFASIS: The Inventory of Factors Affecting Successful Implementation and Sustainment (IFASIS)
GEE: Generalized Estimating Equations
CFIR: Consolidated Framework for Implementation Research
EPIS: Exploration, Preparation, Implementation, Sustainment
NPT: Normalization Process Theory
HEIF: Health Equity Implementation Framework
FQHC: Federally Qualified Health Center
MOUD: Medications for Opioid Use Disorder
PAPERS: Psychometric and Pragmatic Evidence Rating Scale
COSMIN: COnsensus-based Standards for the selection of health Measurement Instruments
C-DIAS: Center for Dissemination and Implementation At Stanford
ATSH: Addiction Treatment Starts Here
SITT-MAT: Stagewise Implementation-To-Target Medications for Addiction Treatment
EMF: Enhanced Monitoring and Feedback
NIATx: Network for the Improvement of Addiction Treatment
RE-AIM: Reach, Effectiveness, Adoption, Implementation, and Maintenance
IMAT: Integrating Medications for Addiction Treatment
ICC: Intraclass Correlation Coefficient
RR: Risk Ratios
