# Correction to: Development and validation of a pragmatic measure of context at the organizational level: the Inventory of Factors Affecting Successful Implementation and Sustainment (IFASIS)

**DOI:** 10.1186/s43058-025-00773-2

**Published:** 2025-08-04

**Authors:** Hélène Chokron Garneau, Hannah Cheng, Jane Kim, Maryam Abdel Magid, Lia Chin‑Purcell, Mark McGovern

**Affiliations:** Stanford Center for Dissemination and Implementation, Department of Psychiatry & Behavioral Sciences, Stanford University School of Medicine, Palo Alto, CA USA

**Correction: Implementation Science Communications 6, 50 (2025)**

**https://doi.org/10.1186/s43058-025-00726-9**

Following publication of the original article [1], the author reported a typographical error in the third sentence of the Abstract: the letter "X" was mistakenly added to the word pooling.

The sentence currently reads:

Existing quantitative measures and the siloed nature of implementation efforts limit possibilities for data poolinXg and harmonization.

The sentence should read:

Existing quantitative measures and the siloed nature of implementation efforts limit possibilities for data pooling and harmonization.
